# Protein evolution in deep sea bacteria: an analysis of amino acids substitution rates

**DOI:** 10.1186/1471-2148-8-313

**Published:** 2008-11-13

**Authors:** Stefano Campanaro, Laura Treu, Giorgio Valle

**Affiliations:** 1 CRIBI Biotechnology Centre, Department of Biology, University of Padua, Via U. Bassi 38/b, 35121, Padua, Italy

## Abstract

**Background:**

Abyssal microorganisms have evolved particular features that enable them to grow in their extreme habitat. Genes belonging to specific functional categories are known to be particularly susceptible to high-pressure; therefore, they should show some evidence of positive selection. To verify this hypothesis we computed the amino acid substitution rates between two deep-sea microorganisms, *Photobacterium profundum *SS9 and *Shewanella benthica *KT99, and their respective shallow water relatives.

**Results:**

A statistical analysis of all the orthologs, led to the identification of positive selected (PS) genes, which were then used to evaluate adaptation strategies. We were able to establish "Motility" and "Transport" as two classes significantly enriched with PS genes. The prevalence of transporters led us to analyze variable amino acids (PS sites) by mapping them according to their membrane topology, the results showed a higher frequency of substitutions in the extra-cellular compartment. A similar analysis was performed on soluble proteins, mapping the PS sites on the 3D structure, revealing a prevalence of substitutions on the protein surface. Finally, the presence of some flagellar proteins in the Vibrionaceae PS list confirms the importance of bacterial motility as a SS9 specific adaptation strategy.

**Conclusion:**

The approach presented in this paper is suitable for identifying molecular adaptations to particular environmental conditions. The statistical method takes into account differences in the ratio between non-synonymous to synonymous substitutions, thus allowing the detection of the genes that underwent positive selection. We found that positive selection in deep-sea adapted bacteria targets a wide range of functions, for example solute transport, protein translocation, DNA synthesis and motility. From these data clearly emerges an involvement of the transport and metabolism processes in the deep-sea adaptation strategy of both bathytypes considered, whereas the adaptation of other biological processes seems to be specific to either one or the other. An important role is hypothesized for five PS genes belonging to the transport category that had been previously identified as differentially expressed in microarray experiments. Strikingly, structural mapping of PS sites performed independently on membrane and soluble proteins revealed that residues under positive selection tend to occur in specific protein regions.

## Background

During the last 30 years, a variety of extremophiles have been isolated from abyssal and hadal environments in diverse locations [1]. These habitats are of particular interest since they are characterized by high hydrostatic pressure, low temperature, lack of nutrients and absence of light. Recently, deep sea adaptation has been investigated by sequencing two genomes of psychropiezophilic bacteria which are considered in this study. These cold and pressure-loving microorganisms are *Photobacterium profundum *strain SS9 [2] and *Shewanella benthica *strainKT99 [3], hereafter called SS9 and KT99. SS9 belongs to the Vibrionaceae family and is a moderately piezophilic, γ-proteobacterium, extensively studied for its importance as a model organism for deep-sea adaptation [4]. It was isolated from an amphipod in the Sulu Sea at 2551 m depth and displays optimum growth at 28 MPa and 15°C. On the other hand, KT99 is a deep-sea obligate piezophile heterotroph, isolated from a sample taken at 9000 m depth in the Tonga-Kermadec Trench in the Pacific Ocean [3]. Both bacteria have a remarkable number of phylogenetically closely-related species that are adapted to shallow water conditions. In order to perform a comparative genome analysis we considered only the species for which complete genomes are available, namely *V. parahaemolyticus*, *V. fisheri, V. vulnificus *for the Vibrionaceae family and *S. baltica*, *S. oneidensis*, *S. frigidimarina for *the Shewanellaceae family, all of which are mesophilic aquatic bacteria. The Shewanella genus can be subdivided into two major branches, the first one characterized by high-pressure adapted species includes *S. benthica *and the other group characterized by pressure-sensitive species contains *S. baltica*, *S. oneidensis *and *S. frigidimarina *[5]. A bioinformatic procedure was applied independently on both families, revealing a valuable number of orthologous genes in each of the two bacterial taxa.

Genetic and biochemical experiments have revealed that both physiological and structural adaptations are essential for high-pressure life. It has been demonstrated that membrane lipids, proteins and solutes accumulation (piezolytes) can influence bacterial growth in deep sea environments [4]. Pressure effects on DNA replication and topology, as well as on cell division, have also been widely discussed as have the nature and regulation of genes that are important for pressure-sensing and the relevance of the transport process in piezophilic bacteria [6].

The object of this study is to further characterize the effect of abyssal conditions on the evolution of SS9 and KT99 genomes. It is not obvious to establish the biological, physical and chemical parameters that are relevant to protein adaptation because abyssal and shallow water environments are extremely different in term of pressure, temperature, light and nutrients. This is an important point to be considered when the two abyssal species described in this paper are compared to phylogenetically related mesophilic species for which our knowledge on their optimal growth conditions is not always fully established. In fact, we are not able to exclude that other parameters, different from hydrostratic pressure, play a role in protein adaptation. Temperature, for example, could influence our analysis as it is one of the parameters that separates *P. profundum *SS9 from the other shallow-water bacteria considered in our comparison. This parameter has probably less influence in shewanellaceae comparison since growth curves determined for shewanellaceae [5] indicate that only *S. oneidensis *can be considered a mesophilic bacterium, whereas *S. benthica*, *S. frigidimarina *and *S. baltica *are psychrophiles. Furthermore all shallow-water bacteria considered in our comparison contain the deoxyribodipyrimidine photo-lyase gene involved in repair of UV radiation-induced DNA damage, whereas the SS9 and KT99 genomes do not code for this enzyme, which is consistent, since there is a distinct absence of sunlight in their normal deep sea environment.

In Table 1 are summarized the main features of the bacteria considered in our study. Growth optimum was obtained from [7] for *P. profundum *SS9, from [8] for *V. parahaemolyticus*, from [5] for *S. baltica*, *S. benthica *e *S. frigidimarina*, while growth data for *S. oneidensis *were kindly provided by Daniel I. of the University Claude Bernard (Lyon).

**Table 1:** Features of the bacteria considered in this paper

Strain	**RefSeq acession no**.	Source	T_opt_	Photolyase	P_opt_	Orthologs
*Photobacterium profundum *SS9	NC_006370	Sulu Sea	15°C	A	28 MPa	Vs
*Vibrio fischeri *ES114	NC_006840	Squid symbiont	30°C	P	ND	3028
*Vibrio parahaemolyticus *RIMD	NC_004603	Osaka	20–43°C	P	0.1 MPa	3469
*Vibrio vulnificus *YJ016	NC_005139	Taiwan	30–40°C	P	ND	3362

*Shewanella benthica *KT99	-	Tonga-Kermadec Trench	4–15°C	A	50 MPa	Vs
*Shewanella baltica *OS155	NC_009052	Baltic Sea	4°C	P	10 MPa	3167
*Shewanella oneidensis *MR-1	NC_004347	Oneida lake	30°C	P	0.1 MPa	2651
*Shewanella frigidimarina *NCIMB 400	NC_008345	North Sea	20–22°C	P	0.1 MPa	2909

Columns report respectively: strain, NCBI accession number, isolation site, optimum growth temperature (T_opt_), presence (P) or absence (A) of the deoxyribodipyrimidine photo-lyase gene, optimum growth pressure (ND = Not Determined) and number of orthologous genes identified for each comparison. The omission of the ORFs encoding light-activated photolyase genes from the SS9 and KT99 genomes is due to the absence of sunlight in the deep sea.

We investigated the presence of positively selected genes in the two bacteria adapted to an abyssal environment, using as a control the phylogenetically related mesophilic species. The computation most frequently used to carry out this analysis is the non-synonymous to synonymous substitution rate ratio, defined omega (ω) [9,10]. In general, an excess of non-synonymous substitutions (dN) over synonymous substitutions (dS) is considered a clear indicator of positive natural selection, because non-synonymous mutations are typically subject to strong purifying selection, whereas synonymous changes are typically neutral.

It should be considered that if we take any two species we would expect to find some genes that underwent positive selection due to the respective evolutionary history of each species. In this study we consider two families of bacteria, each represented by four species. We can assume that some genes underwent positive selection during the evolution of each species, whereas other genes underwent positive selection in clades including more than one species. To identify the genes that underwent positive selection during the evolution of a species we designed the following approach. Firstly, we chose the species for which we wanted to detect genes that underwent positive selection, in our case the deep-sea bacteria. Secondly, we compared the chosen species with the other three control species of the same family, calculating the ω values for each orthologous pair. Thirdly, we performed the calculation of the ω values also within the three control species. Finally, we selected the genes that resulted positive (i.e. underwent positive selection) in each of the three comparisons of the chosen species versus the control species but were negative in the three comparisons within the control species.

It is important to consider that it is likely that the genes selected by this approach specifically underwent positive selection in the chosen species, but this does not automatically imply that they are involved in deep-sea adaptation. This point should be considered for a correct interpretation of the results.

It is known from the literature that evolution of bacterial genes is influenced by several factors, such as protein expression level, functional class and metabolic cost of amino acid residues [11]. Therefore we further considered the distribution of Codon Adaptation Index (CAI) *versus *ω values for each orthologous gene, both in Shewanellaceae and Vibrionaceae. It had been previously defined that CAI is negatively correlated with dN [11]. In fact, proteins expressed at high levels are generally associated with the usage of the "best" synonymous codons, resulting in a direct influence of the codon usage bias on the rate of non-synonymous substitutions in bacteria.

Genome-wide studies have already identified a number of biological processes involved in high pressure adaptation [12]. We used genes that we obtained from the comparison between piezophiles and mesophiles (PS genes) to establish which Gene Ontology (GO) and Cluster of Ortologous Groups (COG) functional classes are targeted by natural selection in bacterial evolution. Moreover PS proteins, belonging to particular categories, can easily show PS sites mainly localized in specific domains. In order to better define the role of amino acid substitutions on deep sea adaptation, we mapped PS sites on the 3D structure as well as on protein topology in respect of trans-membrane regions.

## Results

In order to identify genes under positive selection in deep-sea adapted bacteria we analyzed 4 Vibrionaceae and 4 Shewanellaceae from which we selected 2,174 orthologous genes shared by the former and 2,180 shared by the latter. These numbers are high enough to allow us to explore all the main biochemical and physiological cellular processes. The two groups were obtained by firstly considering the three mesophilic bacteria *V. parahaemolyticus*, *V. fisheri *and *V. vulnificus *for the comparison with *P. profundum *and secondly by using *S. baltica*, *S. oneidensis *and *S. frigidimarina *for the comparison with *S. benthica *(see details in Table 1).

**Figure 1:**
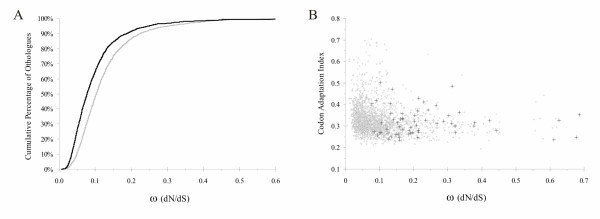
**(A) Cumulative codon rate ratios; (B) distribution of ω values in comparison with CAI**. A) The figure shows cumulative (percentage) codon rate ratios for total Shewanellaceae and Vibrionaceae orthologs. *S. benthica vs. S. frigidimarina *and *P. profundum vs. V. vulnificus *cumulative ω ratios are represented respectively by black and gray lines. B) Distribution of the ω (dN/dS) values computed between the orthologous genes of *S. benthica *and *S. frigidimarina *in comparison with the Codon Adaptation Index calculated for *S. benthica*. The Spearman correlation coefficient (*ρ*) calculated between ω and CAI values is -0.32. PS genes identified in KT99 are highlighted in black. The graph reported is representative of those calculated in all other organisms.

For each orthologous gene pair we aligned DNA sequences and computed amino acid substitution rate. Most of ω values were included in the 0 to 0.2 range (Figure 1A) and the median dN/dS calculated was 0.08 in Vibrionaceae and 0.07 in Shewanellaceae. Using Spearman statistics we verified the inverse correlation between CAI and ω values achieving *ρ *coefficients included in the -0.27 to -0.33 range in Vibrionaceae ortholog comparison and -0.18 to -0.36 in Shewanellaceae (Figure 1B). Moreover PS genes do not have a biased CAI value and their distribution is in accordance with those of all the orthologs. This homogeneous distribution confirms that the ω values of PS genes are not determined by low CAI values but mainly by deep sea adaptation.

It is known that orthologs generally have low ω values (< 0.05), which implies that the proteins are subject to relatively strong purifying selection. Genes with dN/dS > 1 are formally defined as being subject to positive selection, in fact amino acid changes are accumulating faster than would be expected given the underlying silent substitution rate. Even so, proteins with dN/dS < 1 may still contain sites under positive selection, but their contribution to the dN/dS for the whole protein is masked by the purifying selection at other sites [13]. To overcome this problem we only considered the orthologous genes having values significantly higher in the comparison between piezophilic and mesophilic bacteria, with respect to the comparison within mesophiles. Thus, we identified 213 PS genes in the Vibrionaceae family and 61 in Shewanellaceae respectively (see report in Additional files 1, 2). The list includes many different functional classes of proteins, ranging from transporters, that are definitively the most represented, to metabolic enzymes, chaperons and ribosomal proteins.

### Processes Involved in Deep Sea Adaptation

To better understand the role of these genes in the evolution of extremophiles all orthologs were assigned to functional categories according to COG and GO annotations [14,15]. The evidence of PS genes enrichment in COG specific groups was calculated using hypergeometric distribution. Furthermore we used the Fisher exact test on GO in order to give an overview of bacterial adaptation at a higher level of detail, for example specific biological mechanisms. In fact COGs give a more general idea of the processes involved. We obtained two COGs showing evidence of positive selection both in SS9 and KT99: "Nucleotide transport and metabolism" (F) and "Inorganic ion transport and metabolism" (P). There are three additional COG categories only enriched in SS9: "Cell wall/membrane/envelope biogenesis" (M), "Intracellular trafficking, secretion and vesicular transport" (U), "General function prediction only" (R). The "Defense mechanisms" (V) class is KT99 specific, as shown in Table 2. From these data clearly emerges an involvement of the transport and metabolism processes in the deep-sea adaptation strategy of both piezophiles considered in this study, while the adaptation of other biological processes seems to be peculiar to either one or the other.

**Table 2:** COG categories enriched with PS genes in KT99 and SS9

Functional Categories	COGs	KT99	tot	p-value	SS9	tot	p-value
Energy production and conversion	C	5	149	0.214	12	125	0.482
Cell cycle control, cell division, chromosome partitioning	D	1	28	0.175	2	23	0.407
Amino acid transport and metabolism	E	7	173	0.093	22	200	0.261
**Nucleotide transport and metabolism**	***F***	4	48	***0.009***	10	60	***0.032***
Carbohydrate transport and metabolism	G	1	50	0.396	9	91	0.424
Coenzyme transport and metabolism	H	2	114	0.606	4	107	0.987
Lipid transport and metabolism	I	0	82	-	6	65	0.476
Translation, ribosomal structure and biogenesis	J	5	124	0.117	9	125	0.817
Transcription	K	0	126	-	10	136	0.816
Replication, recombination and repair	L	3	107	0.330	5	100	0.945
Cell wall/membrane/envelope biogenesis	**M**	4	106	0.158	16	113	**0.050**
Cell motility	N	1	73	0.596	9	75	0.210
Posttranslational modification, protein turnover, chaperones	O	2	111	0.587	10	102	0.441
**Inorganic ion transport and metabolism**	***P***	4	72	***0.044***	16	107	***0.033***
Secondary metabolites biosynthesis, transport and catabolism	Q	0	34	-	1	28	0.786
General function prediction only	**R**	4	209	0.683	29	217	**0.035**
Function unknown	S	19	659	0.319	52	577	0.789
Signal transduction mechanisms	T	0	105	-	11	116	0.494
Intracellular trafficking, secretion, and vesicular transport	**U**	2	74	0.325	13	81	**0.027**
Defense mechanisms	**V**	3	25	**0.004**	2	36	0.713

**Total Orthologs**		67	2469		213	2484	

Columns represent respectively: functional classes, COG codes, number of PS genes for each category, total number of orthologous genes for each category and p-value calculated using hypergeometric distribution for KT99 (columns 3, 4, 5) and SS9 (columns 6, 7, 8). In **bold **are highlighted significant values (p = 0.05) and the ***italics ***indicate classes enriched in both bacteria.

A similar analysis was performed using the Gene Ontology classification (GO) that takes into account biological processes, cellular components and molecular functions. Both bacterial families reveal an enrichment of PS genes belonging to the "Localization" process, that is the action by which a substance or other structures are transported to (or maintained in) a specific location, see details in Additional file 3. In GO this term is strictly related to "Transport" activity. It is relevant that there are specific "Transport processes" enriched in both piezophiles, instead "Protein folding" and "Cell motility" are present only in SS9. Several genes belonging to the last category are involved in the flagellar basal structure as shown in KEGG representation on Additional file 4[16]. The only significant Cellular Component obtained from the analysis in Shewanellaceae was the "Membrane" category. It emerges also that in Shewanellaceae there is a higher number of enriched categories, but most of them contain only a single gene. For this reason we considered them less noteworthy, even if we cannot exclude that their specific role in the process of adaptation may have been relevant. All genomic analyses were done separately in both bacterial families. This allowed the identification of individual adaptation mechanisms that developed independently in the two extremophiles considered as well as those in common. In fact examining the two lists of PS genes we found only 12 shared by both families, corresponding respectively to 5.6% (12/213) in SS9 and to 18% (12/61) in KT99, see details in Table 3. As a validation of the previous results these genes belong to the most relevant classes: Transport, Membrane and Cell motility. These proteins will be further considered in the Discussion section, due to their highly relevant role in adaptation to these extreme environmental conditions.

**Table 3:** Common orthologous genes identified as positive selected and shared by KT99 and SS9

N°	KT99 Function	Locus Tag	COG	q (%)
**1**	Molybdenum ABC Transporter, Permease Protein	KT99_02056	4149P	10.0
**2**	Hypothetical Protein	KT99_20194	-	0.0
**3**	Phosphoribosylaminoimidazole Carboxylase, Catalytic Subunit	KT99_17021	0041F	0.0
**4**	Dithiobiotin Synthetase	KT99_10643	0132H	4.9
**5**	Uracil Permease	KT99_16519	2233F	0.0
**6**	TonB2 Protein	KT99_09573	0810M	10.0
**7**	Primosomal Replication Protein N, Putative	KT99_00710	-	7.0
**8**	Methylated DNA-Protein Cysteine Methyltransferase	KT99_15170	0350L	7.0
**9**	NADH Dehydrogenase	KT99_13367	1252C	0.0
**10**	Succinate-Semialdehyde Dehydrogenase	KT99_19874	1012C	0.0
**11**	MSHA Biogenesis Protein MshL	KT99_07703	1450NU	10.0
**12**	RND Multidrug Efflux Transporter MexF	KT99_04334	0841V	10.0

**N°**	**SS9 Function**	**Locus Tag**	**COG**	**q (%)**

**1**	Putative Phosphate ABC Transporter, Permease Protein	PBPRA1392	0581P	4.3
**2**	Hypothetical Protein	PBPRA2020	-	4.8
**3**	Hypothetical Phosphoribosylaminoimidazole Carboxylase, Catalytic Subunit	PBPRA3574	0041F	0.0
**4**	Dithiobiotin Synthetase	PBPRA2326	0132H	8.3
**5**	Putative Xanthine/Uracil Permease	PBPRA0186	2233F	8.3
**6**	Hypothetical TonB Protein	PBPRA2103	0810M	4.4
**7**	Hypothetical Primosomal Replication Protein N	PBPRA1010	-	8.3
**8**	Hypothetical Methylated DNA-Protein Cysteine Methyltransferase	PBPRB0210	0350L	4.3
**9**	Putative Nitrite Reductase (NAD(P)H), Large Subunit	PBPRA1428	1251C	8.3
**10**	Putative Succinylglutamate 5-Semialdehyde Dehydrogenase	PBPRA0291	1012C	3.3
**11**	Hypothetical Flp Pilus Assembly Protein	PBPRA2496	4964U	3.3
**12**	Putative Multidrug Resistance Protein	PBPRA2721	0841V	4.3

In the first column orthologs are ordered correspondingly in the two families. The NCBI annotation, locus tag, COG categories and SAM significance q-value are reported in columns 2–5. q-value is obtained from SAM software and it is the lowest False Discovery Rate at which the gene is called significant.

### Localization of PS Sites on Protein Structure

For a better comprehension of the variable amino acids functional role we mapped them on protein structures, domains and trans-membrane regions. We identified amino acids specifically different from a chemical-physical point of view in piezophiles, compared to mesophiles. Hereafter we will call them PS sites.

**Figure 2:**
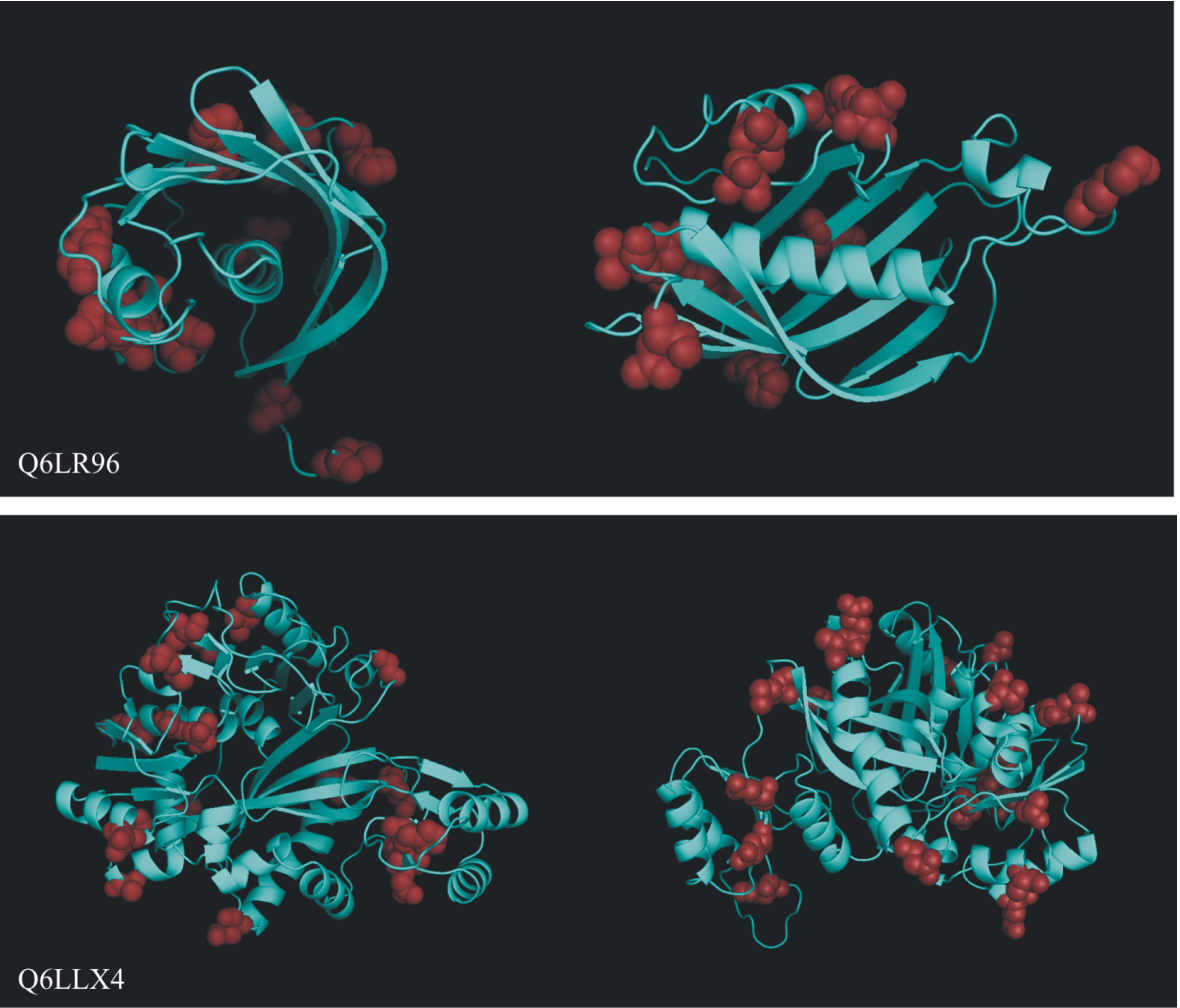
**Distribution of PS sites on two protein structures**. Modeled structure of Q6LR96 protein (3-hydroxydecanoyl-ACP dehydratase) and of Q6LLX4 (phosphoribosylamine-glycine ligase) coded respectively by *P. profundum *SS9 PBPRA1773 and PBPRA3420 genes. Sites showing evidence of positive selection (P < 0.01) are depicted as red spheres. From two different perspectives it appears that most of PS sites are located on the protein surface.

Position analyses were done using two distinct strategies for soluble and membrane proteins, due to the different meaning of amino acid substitutions in these categories. Among the 213 PS proteins identified in Vibrionaceae family, 65 of them have predicted trans-membrane regions and 149 are predicted as soluble. By querying the ModBase database [17] we obtained 42 models having more than 40% similarity with known protein structures. Using these models we mapped the PS sites on the 3D structure of soluble proteins using PyMol software (Figure 2).

Generally the variable amino acids are localized on the protein surface. To confirm their distribution, we evaluated the number of solvent exposed PS sites (Additional file 1). This computation was performed for all SS9 models with the exception of PBPRA0158, as it is trans-membrane.

**Figure 3:**
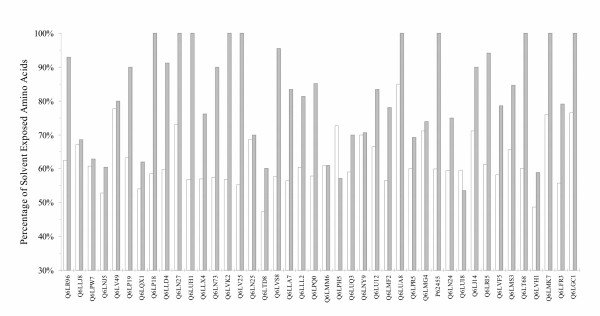
**Percentage of solvent exposed PS sites for soluble proteins**. In this histogram are reported the percentages of solvent exposed amino acids (white columns) and those of solvent exposed PS sites (grey) for each modeled protein. All values were calculated for SS9 proteins.

In 27 proteins more than 75% of the PS sites are located on the surface while in other 14 cases they fall in the 50% – 75% range. We verified that the variable amino acids are predominantly solvent exposed, as reported in Figure 3. Moreover, considering the total amino acid number, we found 4 proteins with more than 75% of solvent exposed residues, 37 fell in the 50% – 75% range and one had less than 50% exposed residues. All these results indicate that in only three cases the fraction of solvent exposed PS sites is lower or equal to the one calculated for the entire structure. Finally, in nine cases we detected PS sites involving ligand binding sites.

**Figure 4:**
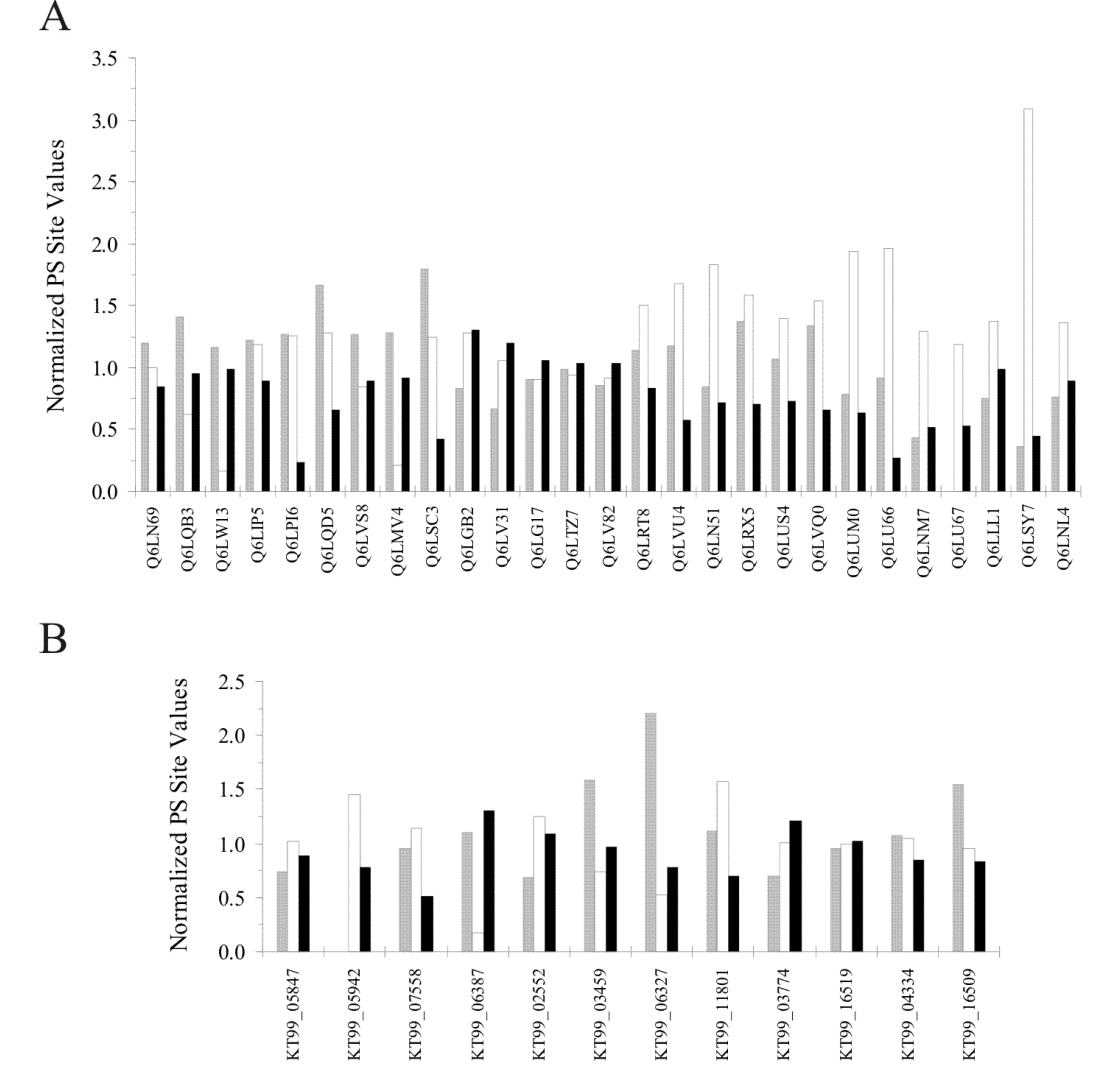
**Distribution of PS with respect to membrane protein topology**. The normalized fraction of trans-membrane PS sites (black columns) is compared to the cytoplasmic (grey) and external (white) PS sites. Results are shown only for Vibrionaceae (A) and Shewanellaceae (B) transporters. KT99 TrEMBL IDs are not available, for this reason in (B) are reported NCBI's Locus Tags.

In the analysis of the PS sites of membrane proteins, particular relevance was given to their position. We looked at protein topology, inside or outside the membrane, and at trans-membrane helices. We found 21 proteins with a higher number of PS sites in trans-membrane region, 18 in the external region and 22 in the cytoplasmic region. However these numbers are heavily dependent on the correspondent region length and normalization was required to obtain comparable results between different proteins. The outcome indicates that the higher fraction of PS sites is externally located in 41% of proteins, cytoplasmic in 35% and trans-membrane in 24% (Figure 4A). Moreover, comparing membrane PS sites with extracellular and cytoplasmic together, only 33% of the proteins had the higher variable amino acids in the trans-membrane helices. Finally, we calculated the mean number of cytoplasmic, external and trans-membrane PS sites normalized with respect to each portion length obtaining respectively 1.03, 1.06 and 0.86. Due to their relevance in our study, we also executed the same procedure considering only transporters, in this way we got higher mean values, respectively 1.02, 1.28 and 0.77 for cytoplasmic, external and trans-membrane regions. Among the PS genes of Shewanellaceae we could only analyze 12 trans-membrane proteins because no 3D model of soluble proteins was available in ModBase. Despite the low number of genes, structural mapping of PS sites on trans-membrane proteins revealed that the residues under positive selection occur preferentially in the extracellular region (5 proteins), as shown in Figure 4B. Calculating the mean number of normalized cytoplasmic, external and trans-membrane PS sites, we obtained respectively 1.05, 0.99 and 0.91.

It has to be considered that only some portions of the proteins are under positive selection and this influences the ω value computed for the entire sequence. We searched for genes under intensive selective pressure to recognize regions with overrepresented nonsynonymous mutations unevenly distributed along the alignment, previously unidentified using conventional rate comparison analysis [18]. We were interested in genes with overabundant mutation regions localized in specific domains that were present in all the comparisons between piezophiles and mesophiles, but absent in mesophilic bacteria only. We discovered just one fast evolving region (FER) in PBPRA0616 among all Vibrionaceae orthologs and we found 10 genes with overabundant mutation rate (OMR). Four of these are trans-membrane proteins and six are soluble proteins. In KT99 we found one FER in the KT99_15170 gene, whilst one trans-membrane protein and five soluble proteins have OMRs (Additional files 1 and 2).

Finally we investigated if the SS9 PS proteins had been previously identified as involved in high pressure and low temperature adaptation and we compared our data with SS9 microarray experiments results [6] and with genes found by systematic mutagenesis screening [19]. Among the 14 PS genes found differentially expressed by microarray analyses, 4 of them were identified as up-regulated under high hydrostatic pressure (28 MPa) compared to atmospheric pressure (0.1 MPa) and 7 were found to be down-regulated; 2 were down-regulated at 4°C compared to 16°C. Two genes, PBPRA2089 and PBPRA0281, were identified in the list from the systematic mutagenesis screening as responsible for piezosensitivity in SS9 mutants.

## Discussion

Vibrionaceae and Shewanellaceae represent a significant portion of the culturable heterotrophic bacteria of oceans, coastal waters and lakes. Some species belonging to these families also inhabit deep-sea environments and, due to the increasing interest in high pressure adaptation, various studies have been undertaken to clarify their characteristics from a genomic point of view [12]. Our study is based on an exhaustive comparison of orthologous genes. It is therefore important to discuss the criteria used for their selection. Two main factors affect the number of orthologous genes shared amongst the considered species: the number of species and their phylogenetic distance. Therefore, we kept the two bacterial families separated and selected two independent sets of orthologous genes, one for each of the two families. Furthermore, for each family we limited the number of species to four: one from deep sea and three from shallow water. In both families we obtained about 2,180 shared orthologous genes. The general rationale of our study is to identify, independently for each family, the genes that underwent positive selection in the deep-sea adapted species. Thus, using Gene Ontology associations, we should be able to see whether any biological processes or molecular functions are particularly affected in one or both bacterial families.

### PS Genes Detection

In both families about 2180 ortologues were identified and utilized in six different two by two ω value calculations, three between piezophiles-mesophiles and three between mesophiles, the latter considered as a negative control. The level of divergence among the species of a bacterial family is high enough to detect the signature of selection, yet low enough to have a high number of orthologous genes. In previous works computations performed using the dN/dS substitution rate were complicated by the uncertainty of the threshold to be considered for genes under positive selection. For this reason, instead of a direct selection of all the instances above a given threshold, we decided to consider only those genes that had statistically higher ω values in the three piezophile-mesophiles comparisons. The approach employed allowed us to identify both specific and common functional categories involved in SS9 and KT99 adaptation strategies. In fact, both of them are piezophiles but they were isolated from distinct environments and showed optimum growth at different pressures and temperatures.

### Functional classes

To clarify the role of PS genes we analyzed their statistical over-representations in all functional classes. As expected evaluation of both COG and GO results confirmed the key role of transport proteins in abyssal adaptation. It is known from the literature that the transport of compounds like Trp, Lys, His and Leu is reduced at high pressure, owing to the volume change of activation needed in the transport process [2]. In fact, the *tat2 *gene enables transformed yeast cells to grow at high hydrostatic pressure by encoding a high-affinity tryptophan permease which counteracts the significant inhibition of tryptophan uptake into the cells due to high pressure [20]. It was previously determined that SS9 up-regulates certain transporters mainly at low pressure. This could indicate that these proteins may have evolved a particular structure adapted to elevated pressure because their up-regulation could compensate the reduction of functionality.

Five PS genes previously identified as differentially expressed in microarray experiments probably play a crucial role (Additional file 1). Among these, there are three permease components that are part of an ABC transporter: "hypohetical ABC transporter, permease protein" (PBPRA2115); "putative ABC-type arginine transport system, permease component" (PBPRA2740); "putative ABC-type metal ion transport system, permease component" (PBPRA2941). The remaining are a "periplasmic peptide binding protein" belonging to a putative peptide ABC transporter (PBPRA0525) and a "putative glycerol-3-phosphate transporter" (PBPRA0158). PBPRA0525 is part of the ABC transporter coded by PBPRA0521-PBPRA0525 that was previously shown to be up-regulated at 28 MPa and 4°C, while a second peptide ABC transporter, coded by PBPRA2934-PBPRA2938 genes, was down-regulated at 28 MPa. These data indicated that SS9 can express proteins adapted to different pressures [6]. The fact that only PBPRA0525 was identified in our study is a further indication that this protein has been selected to work better in the abyssal environment, and gives indirect evidence that SS9 probably evolved from some mesophilic strains.

Moreover, the number of PS sites in the piezophile permease compared to those of the mesophiles is twice (66/559 *versus *30/608) in PBPRA0525, which is over expressed at high pressure and low temperature.

Another class of transporters that are influenced by pressure is that of the voltage-gated channels-. Using the patch clamp technique, it has been demonstrated that pressure acts on the movement of the charge sensor and on the conformational change involved in opening the channel pore [21-23]. For certain channels the ΔV value for the closed/open transition is -105 ml mol^-1^, that is, the open state occupies a smaller volume than the closed state [24] thus implying that pressure could have a large influence on this process. In this work we verify that in deep-sea organism transporters there are PS sites, a finding that had previously been hypothesized -by other authors [21]. -^-^In light of these points the demonstration that many PS genes belong to the GO transport category is noteworthy. Despite the high number of different classes of transporters made difficult to discuss the role of the localization of the PS sites on protein structure, their high frequency in the external part of the membrane proteins is noteworthy.

Another significant group of proteins is that involved in protein export across the outer membrane [25]. Two of them, "protein export protein SecD" (PBPRA0745) and "protein export protein SecF" (PBPRA0746), belong to the translocation complex and were identified as positive selected (Additional file 4). SecDF of *B. subtilis *is implicated in early translocation steps, whereas in *E. coli *these polypeptides are required to release mature proteins. It was found that strains lacking intact *secDF *are cold-sensitive and this conditional phenotype probably reflects the thermal sensitivity of protein translocation [26]. As previously noted high pressure and low temperature have a similar effect on bacterial membranes and are probably responsible for positive selection acting on these two proteins and globally on SS9 transporters [27]. The maintenance of biological membranes in a narrow range of viscosity, homeoviscous response, or within a liquid-crystalline phase, homeophasic response, is essential for growth and survival [28,29]. This is not surprising, because a great number of biological processes such as membrane transport, protein-protein interactions within the lipid bilayer, metabolic electron transport, intracellular signaling and gene regulation are dependent on a suitable membrane physical structure [30]. Due to the key role played by membrane proteins, we computed the distribution of PS sites in different domains; however, our analysis showed that in 40% of the proteins, considering normalized values, the higher fraction of PS sites is located in the external part and not in trans-membrane region, the opposite of what was expected considering the putative influence of the membrane composition mentioned above. This finding is in agreement with similar results obtained in mouse, where, compared with the intracellular (cytoplasmic and nuclear) domains, a greater proportion of extracellular domains possess higher ω values [13] and are subject, on average, to greater positive diversifying selection. Moreover other studies comparing transporters of Bacteria, Archea and Eucarya [31] indicate that the major differences in terms of size between orthologs are in the hydrophilic regions, the most important from a regulatory point of view.

Some of the SS9 PS orthologs we identified belong to the GO categories related to the following terms: "Locomotion", "Ciliary or flagellar motility" and "Cell motility" (Additional file 3). This is probably related to the importance of bacterial motility in enhancing bacteria-organic-matter coupling and reducing the ability of protozoa to graze on bacteria [32]. It was shown that the *P. profundum *deep sea adapted strains (SS9 and DSJ4) contain an additional gene cluster absent in the shallow bathytype (3TCK) which resembles genes for the production of lateral flagella [6]. The function of this additional cluster is unknown, but preliminary results have provided evidence that the SS9 strain displays high motility at 28 MPa pressure and swims very slowly at atmospheric pressure, while the opposite phenotype has been observed in the shallow water 3TCK [33]. Proteins coded by the PS genes, which we recognized as part of this pathway, are the "putative flagellar basal-body rod protein FlgF" (PBPRA0905) and the "putative polar flagellar FlgG" (PBPRA0906) located on the external membrane region. Also involved in this process are the "flagellar basal body-associated protein FlgB" (PBPRA0901) and the "flagellar basal body protein FliH" (PBPRA0925), both part of the motor switch (Additional file 4). Flagellar assembly and motor function are known to be the most pressure-sensitive cellular processes [4,34] therefore we hypothesized this modification of flagellar structure to be a strategy specific to SS9. In fact in the KT99 PS gene list there are no flagellar proteins and the only one related to the cell motility class is "MSHA biogenesis protein MshL" (KT99_07703), part of the Type II secretion system.

We have already stated that independent analysis done on Shewanellaceae and Vibrionaceae allowed the detection of functional classes involved in SS9 and KT99 specific and common adaptations. In order to further investigate the most interesting pathways we compared the two PS gene lists using BLAST and we found 12 genes shared by them (Table 3). They are respectively 18% and 5.6% of the corresponding total of PS genes. Among the proteins coded by them there are three transporters and this stresses their importance in deep-sea adaptation.

The presence of the "hypothetical Primosomal Replication Protein N" is relevant as it has a role in the assembly of the primosome and has a helicase activity [35]. The same function is present in RecD protein of SS9 that also enhances the pressure resistance in *E. coli *[36].

Positive selection acting on this protein could counteract the reduction in DNA and RNA biosynthesis at high pressure in mesophiles but also the inhibition of cellular division that could determine the formation of highly filamentous cells [37,38].

### Localization of PS sites in the Protein Structure

It has been proposed that solvent exposed amino acids have a lower purifying selection and for this reason could vary more easily. An explanation could be the lower functional constraint of the residues located on the protein surface with respect to the buried residues. For most of the proteins this is clearly evidenced from the PhyMol software generated images (Figure 2). To verify this assumption we computed the fraction of solvent exposed PS sites in 41 SS9 proteins for which a modeled structure was available in database. Comparing the fraction of the solvent-exposed amino acids with that of the PS sites, we found the latter higher in 93% of the proteins. Despite the higher tendency of the exposed amino acids to change, we could hypothesize that protein surface has a role in deep-sea adaptation. For example [39] it has been reported that the increased exposure of hydrophobic residues and the reduction of solvent exposed charged amino acids determines a destabilization of the protein surface. This is linked to cold adaptation, reduction of activation energy and to an increased catalytic efficiency [40,41].

## Conclusion

In this paper we propose a new statistical approach that could be applied to identify genes under positive selection by means of omega value calculation. Using this method we investigated the problem of molecular adaptation in deep-sea adapted bacteria. Genes obtained in vibrionaceae and shewanellaceae were grouped using COG and GO and the results showed that positive selection in piezophiles targets a wide range of different functions such as transport of solutes, protein translocation, DNA synthesis and flagellar motility. Transport was identified as the most notable biological process that underwent positive selection in the two deep-sea bacteria that we studied. This result is particularly interesting because it confirms some previous findings obtained by other approaches such as the analysis of expression profiles at high and low pressure [2] and the analysis of laterally transferred genes in deep-sea bacteria [6]. We can therefore conclude that the proteins involved in transport represent a bona fide class of proteins that requires consistent modifications to adapt to the deep-sea environment.

Structural mapping of PS sites in membrane proteins reveals that they tend to occur preferentially in extracellular regions of membrane proteins.

## Methods

Among the completely sequenced bacterial genomes deposited in the NCBI database [42], we chose those with the larger number of orthologous genes with respect to the two piezophiles that we wanted to investigate. To select these microorganisms all protein-coding gene sequences in SS9 and KT99 were blasted -^-^respectively against the Vibrionaceae and Shewanellaceae available at NCBI's ftp site [43]. BLAST results were parsed using PERL scripts in order to select only those orthologs satisfying alignment criteria defined in literature [14]: alignment extension over 70% and e-value cut-off 10^-5^. All identity values were higher than 40% and most of e-value indicated an exact match. To discard paralogous genes from our analysis, reciprocal BLAST searches were performed and the selection was limited at best reciprocal hits. Then we selected the definitive orthologous genes choosing those of the three mesophilic bacteria with the higher number of matches in the pair comparison with the piezophile. Only SS9 or KT99 proteins with a valuable scoring match in each of the three mesophilic bacteria were considered. All the following procedures were carried out separately for Shewanellaceae and Vibrionaceae families.

### Tests for positive selection

The positive selection analysis was performed considering all the possible pairs of bacteria and six ω values series were calculated. This kind of test required a specific DNA alignment for each gene based on the corresponding protein alignment. For this reason, orthologous protein sequences were aligned with ClustalW [44] and then were back-translated to the corresponding DNA sequences using RevTrans software. This allowed the preservation of gaps obtained during protein alignment, essential to avoid in-dels causing frameshifts in DNA sequences.

To test for positive selection each ortholog pair of the six series was compared using the method implemented in *yn00 *software of PAML package [45] that evaluates the presence of PS sites calculating the dN/dS ratio (ω) between sequences pairs. The results were six groups of values: three from the comparison of the piezophilic bacteria with the mesophiles and three from the mesophilic bacteria between them.

### Codon Adaptation Index (CAI)

CAI values for orthologous genes were obtained for all bacteria using CodonW [46]. This software performs correspondence analysis of the Relative Adaptiveness (RA) of the codon usage of a gene towards the codon usage of highly expressed genes. RA of each codon is the usage ratio with respect to that of the most abundant codon for the same amino acid. This estimate was done selecting genes identified in SS9 and *S. oneidensis *MR-1 from those having high expression values (fluorescence arbitrary units higher than 10000) in microarray experiments performed on these bacteria. SS9 microarray results were obtained from ArrayExpress database (E-MEXP-210) [47], instead *S. oneidensis*'s ones where taken from GEO database (GSM100358, GSM100396, GSM100872, GSM100873, GSM87832, GSM88468, GSM88473, GSM88474) [48].

### SAM analysis

The six values reckoned for each ortholog were therefore analyzed in order to identify those significantly higher in the comparison between piezophilic and mesophilic bacteria with respect to the values obtained from the mesophiles only. These genes could possibly be affected by positive selection in piezophiles (PS genes) and could play a role in bacterial adaptation to deep-sea environment.

Statistical analysis was performed using SAM [49]. This software was initially developed for microarray data analysis and implements a modified t-test useful to verify large number of independent hypothesis. SAM employs repeated permutations to settle which of the values obtained in the piezophile-mesophiles comparisons are significantly higher than those identified comparing mesophiles only. The cutoff for significance is determined by a tuning parameter delta, chosen by the user and based on the false discovery rate (FDR) set at 10%. All the following bioinformatics analyses were executed on PS genes lists obtained as described above.

### COG and GO functional classes

PS genes were evaluated to identify enrichment of GO and COG categories relative of what would have been expected by chance alone.

While GO enrichment was calculated using GoMiner software [50], the COG calculation was done according to the hypergeometric distribution. With this analysis we identified the chance of observing the number of genes annotated with a particular COG category among the selected group of orthologs. The probability P of finding at least *k *genes of a functional category within a group of *n *genes is given by:

$$P=\sum_{i=k}^{n}\frac{\left(\begin{array}{c} f\\ i \end{array}\right)\left(\begin{array}{c} g-f\\ n-i \end{array}\right)}{\left(\begin{array}{c} g\\ n \end{array}\right)}$$

where *f *is the total number of genes within the same category (in the matrix) and *g *is the total number of genes identified using SAM software. Hypergeometric distribution was calculated using R statistical package [51] and the significance threshold considered is 0.05.

### Fast evolving regions prediction

A variant of the TL method [52] was used on the PS genes to detect regions with significantly higher variability within each sequence pairs. The number of nonsynonymous mutations is estimated on every codon position in the alignment and regions with overabundant mutation rate are reckoned using Faster software [18]. These sections were further selected and considered in the study only if present in piezophile-mesophiles comparisons and discarded if also identified comparing mesophiles.

### PS sites mapping on 3D structures of proteins in Vibrionaceae

When available, we downloaded from ModBase the 3D structure PDB file calculated by comparative modeling for each PS gene [53]. To get more reliable results we considered only the proteins with a percentage of identical residues higher than 40% in the alignment between the target and the template. For each ortholog we aligned the three mesophile sequences first independently and then with the piezophile sequence using ClustalW. In this way we identified amino acids conserved among the three but variable with respect to the piezophile sequence. We considered as variable only those amino acids having strongly different physical-chemical properties. These sites, along with the position of the ligand binding site downloaded from ModBase, were highlighted in the 3D protein structure using PyMol software. We also mapped in the structure the position of the overabundant mutation region, when present. Then the DSSP software was utilized to estimate solvent accessible surface areas (ACCs) for all residues of each ortholog PDB file selected above [54]. Finally we compared each protein mean ACC value with those obtained only for the PS sites.

### PS sites localization on trans-membrane proteins

For each selected membrane protein we estimated the trans-membrane helices positions and the protein orientation, as predicted by Phobius software [55]. Therefore we localized the PS sites position in all sequences and we mapped them with respect to the protein topology, inside or outside the membrane, and on trans-membrane helices. For each of these sections the number of PS sites was normalized for their total number and the protein region length. The values for exposed (ext), trans-membrane (tr) and cytoplasmic (cyt) PS sites are given by:

(ext N° of PS sites/tot N° of PS sites)/(ext N° of AA/tot N° of AA)

(cyt N° of PS sites/tot N° of PS sites)/(cyt N° of AA/tot N° of AA)

(tr N° of PS sites/tot N° of PS sites)/(tr N° of AA/tot N° of AA).

## Authors' contributions

SC conceived of the study, performed the statistical and Gene Ontology analysis, participated in the interpretation of the results and drafted the manuscript.

LT participated in the design of the study, developed the PERL scripts, calculated the omega values, participated in the interpretation of the results and drafted the manuscript.

GV participated in the design and in the coordination of the study and revised the manuscript. All authors read and approved the final manuscript.

## Supplementary Material

Additional file 1 **Additional information on PS genes identified in Vibrionaceae family**. In columns A-H are reported respectively: internal database ID, NCBI Locus Tag, TrEMBL ID, NCBI gene annotation, COG category, statistic q-value, total number of amino acids and of PS sites for each *P. profundum *SS9 orthologue. The ω values obtained with PAML software for the six pair-comparisons and CAI values calculated for each bacterium are reported in I-R; percentage of total solvent-exposed amino acids and solvent-exposed PS sites are shown in columns S and T. In the table are also highlighted differentially expressed genes identified in SS9 microarray experiments (yellow background), common PS genes between SS9 and KT99 (underlined), piezosensitive mutants (italic), genes with overabundant mutation region (red), and proteins with modeled 3D structure (bold). Organism names were abbreviated as follows: *P. profundum *SS9 (P), *V. vulnificus *(V1), *V. parahaemoliticus *(V2) and *V. fisheri *(V3). Click here for file

Additional file 2 **Additional information on PS genes identified in Shewanellaceae family**. In colums A-H are reported respectively: internal database ID, NCBI Locus Tag, TrEMBL ID, NCBI gene annotation, COG category, statistic q-value, total number of amino acids and of PS sites for each S. benthica KT99 orthologue. All reported TrEMBL IDs and COG annotations refer to *S. oneidensis *MR1. The ω values obtained with PAML software for the six pair-comparisons and CAI values calculated for each bacterium are reported in M and N. In the table are also highlighted common PS genes between SS9 and KT99 (underlined) and genes with overabundant mutation regions (red). Organism names are abbreviated as follows: *S benthica *KT99 (P), *S. oneidensis *(S1), *S. frigidimarina *(S2) and *S. baltica *(S3). Click here for file

Additional file 3 GO categories enriched with PS genes in Shewanellaceae and Vibrionaceae*. Columns represent respectively: functional classes, GO codes, number of PS genes for each category, total number of orthologous genes for each category and p-value calculated using GoMiner software for Shewanellaceae (columns 3, 4, 5) and Vibrionaceae (columns 6, 7, 8). In bold are highlighted the significant values and the gray background indicates the enriched classes both in Shevanellaceae and Vibrionaceae. The asterisk indicates that the functional class names have been shortened, please refer to the GO code to recover the correct annotation term. Click here for file

Additional file 4 **KEGG schematic representation of Flagellar assembly (A) and Protein export Sec pathway (B)**. PS genes of SS9 were highlighted in blue on KEGG pathways and modules. As discussed in the text both motility and transport contain a high number of PS genes. Click here for file
