# Supplementary material for: Protein evolution in deep sea bacteria: an analysis of amino acids substitution rates
Source: BMC Evol Biol. 2008 Nov 13;8:313. doi: 10.1186/1471-2148-8-313 (PMC2600651; doi:10.1186/1471-2148-8-313)
Supplement: Additional file 4 — KEGG schematic representation of Flagellar assembly (A) and Protein export Sec pathway (B). PS genes of SS9 were highlighted in blue on KEGG pathways and modules. As discussed in the text both motility and transport contain a high number of PS genes. [file 1471-2148-8-313-S4.doc]

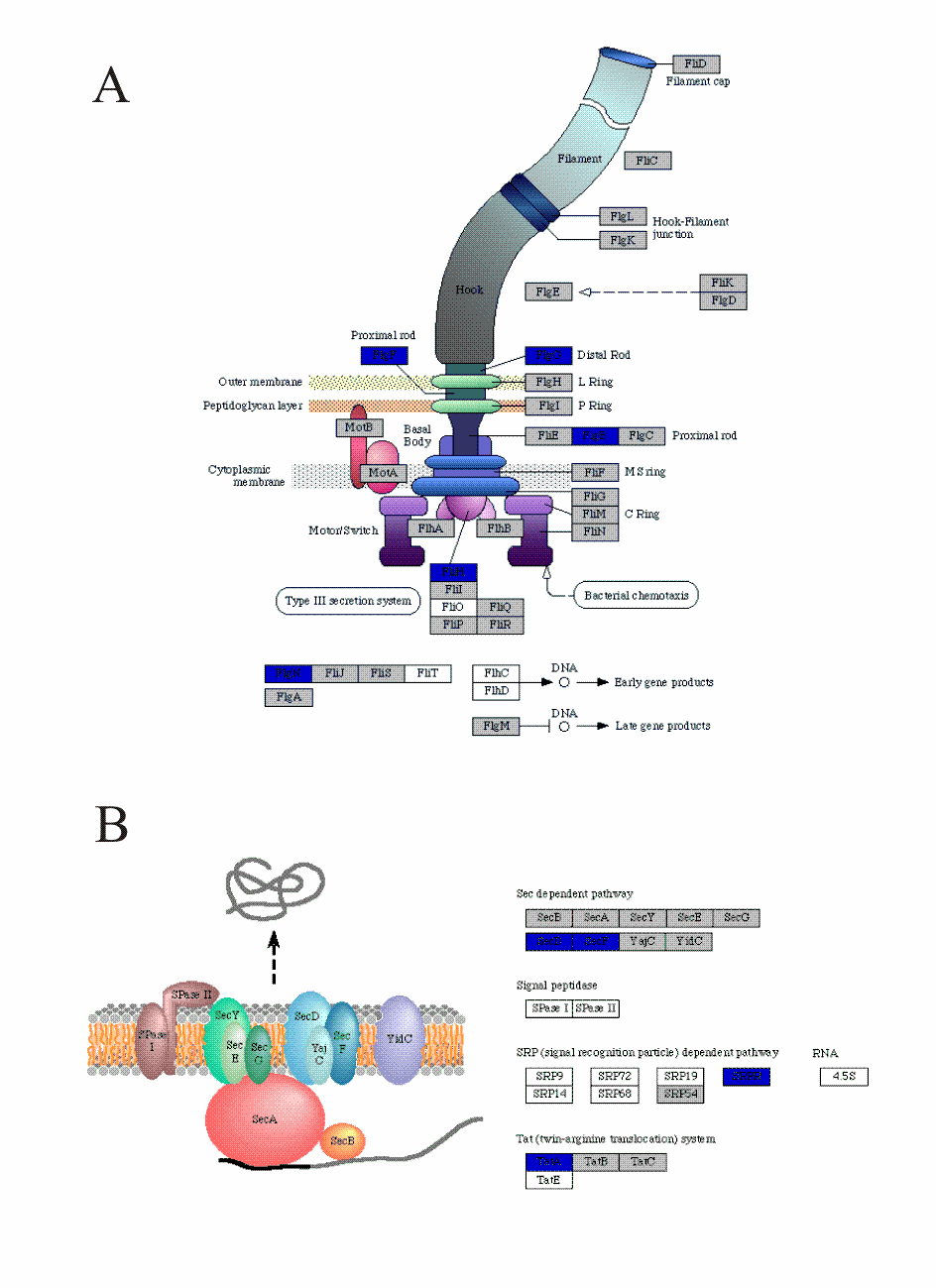


**Additional file 4. KEGG schematic representation of Flagellar assembly (A) and Protein export Sec pathway (B). PS genes of SS9 are highlighted in blue.**
